# Authors’ Response

**DOI:** 10.1308/rcsann.2014.175

**Published:** 2014-03

**Authors:** D Hariharan, DN Lobo

**Affiliations:** Nottingham University Hospitals NHS Trust, UK

We thank Dr Steelman for her interest in our article and for her comments. We agree with her that distraction and multitasking in the process of counting sponges and instruments in the operating theatre leads to incorrect counts. The distractions result from prolonged operating time, multiple personnel in the operating room, change of personnel while the surgical procedure is being performed, operations performed as an emergency and surgical procedures performed out of hours where the number of personnel may be few, requiring them to multitask. These significant predictors of count discrepancy were listed in Table 4 of our manuscript.

Dr Steelman has raised some concerns with the algorithm we proposed in Figure 2. We would, however, like to reiterate that we have clearly stated that in the event of a count discrepancy in the operating theatre, it is the responsibility of the lead surgeon to perform a thorough body cavity search to look for the missing item. If the discrepancy persists despite a thorough body cavity search, radiological imaging should be sought to resolve the discrepancy even if it means transferring the patient outside of theatre. The implications of retained sponges or instruments were discussed in detail in our paper, and the additional expense (and return to theatre if necessary) remain justified.

We also thank Dr Steelman for highlighting a new study evaluating the incorporation of a radiofrequency detection system into existing laparotomy sponge counting protocols for the detection of retained sponges and defining associated risk factors for the same.^1^ We reviewed the English language literature published between January 2000 and June 2012, and as the paper referred to was published in October 2012, it was outside the review period. We recognise that the human effort of counting sponges and instruments is prone to error, and an urgent need exists to incorporate and evaluate new technologies that could be used to assist prevalent methods to reduce rates of retained objects; in our article, we discussed the merits of the adjunctive use of sponges tagged with radiofrequency identification chips.

## References

[1] Rupp CC, Kagarise MJ, Nelson SM (2012). Effectiveness of a radiofrequency detection system as an adjunct to manual counting protocols for tracking surgical sponges: a prospective trial of 2,285 patients. J Am Coll Surg.

